# The Applicability of Standard Error of Measurement and Minimal Detectable Change to Motor Learning Research—A Behavioral Study

**DOI:** 10.3389/fnhum.2018.00095

**Published:** 2018-03-22

**Authors:** Leonardo Furlan, Annette Sterr

**Affiliations:** ^1^Brain and Behaviour Research Group, School of Psychology, Faculty of Health and Medical Sciences, University of Surrey, Guildford, United Kingdom; ^2^Neurology Clinical Division, Clinics Hospital, São Paulo University, São Paulo, Brazil

**Keywords:** motor learning, neurorehabilitation, plasticity, inferential statistics, *p*-value, reliability, standard error of measurement, minimal detectable change

## Abstract

Motor learning studies face the challenge of differentiating between real changes in performance and random measurement error. While the traditional *p*-value-based analyses of difference (e.g., *t*-tests, ANOVAs) provide information on the statistical significance of a reported change in performance scores, they do not inform as to the likely cause or origin of that change, that is, the contribution of both real modifications in performance and random measurement error to the reported change. One way of differentiating between real change and random measurement error is through the utilization of the statistics of standard error of measurement (SEM) and minimal detectable change (MDC). SEM is estimated from the standard deviation of a sample of scores at baseline and a test–retest reliability index of the measurement instrument or test employed. MDC, in turn, is estimated from SEM and a degree of confidence, usually 95%. The MDC value might be regarded as the minimum amount of change that needs to be observed for it to be considered a real change, or a change to which the contribution of real modifications in performance is likely to be greater than that of random measurement error. A computer-based motor task was designed to illustrate the applicability of SEM and MDC to motor learning research. Two studies were conducted with healthy participants. Study 1 assessed the test–retest reliability of the task and Study 2 consisted in a typical motor learning study, where participants practiced the task for five consecutive days. In Study 2, the data were analyzed with a traditional *p*-value-based analysis of difference (ANOVA) and also with SEM and MDC. The findings showed good test–retest reliability for the task and that the *p*-value-based analysis alone identified statistically significant improvements in performance over time even when the observed changes could in fact have been smaller than the MDC and thereby caused mostly by random measurement error, as opposed to by learning. We suggest therefore that motor learning studies could complement their *p*-value-based analyses of difference with statistics such as SEM and MDC in order to inform as to the likely cause or origin of any reported changes in performance.

## Introduction

In motor learning studies, investigators typically assess individuals for their performance on a motor task before, during, and after a period of training on the same task (e.g., Pascual-Leone et al., 1994; Karni et al., 1995; Reis et al., 2009; Debas et al., 2010; Abe et al., 2011; Platz et al., 2012a,b). One of the challenges in such studies is to differentiate between real changes in performance and random measurement error. The latter corresponds to changes that occur at random in performance scores, as opposed to, for instance, changes due to learning. Potential sources of random measurement error include, among others: (i) differences in individual factors such as level of motivation, fatigue, attention, etc. at different test sessions, (ii) an intrinsic variability of the measurement instrument or test employed to measure performance, or (iii) a combination of both (Beninato and Portney, 2011; Portney and Watkins, 2015). In fact, any measurable change in motor performance is likely to be a compound of both real modifications in performance and random measurement error, each contributing at varying degrees to the observed change (Beninato and Portney, 2011; Portney and Watkins, 2015).

Research on motor learning and its enhancement in humans is relevant to many fields of applied research. It can contribute for instance to improve learning in sports, music, industry, and medical training (Schmidt and Lee, 2014), and has also been extensively linked to sleep research (Landmann et al., 2014). Another relevant application includes the optimization of (re)learning in patients undergoing physical rehabilitation, for example, after brain damage such as stroke (Dayan and Cohen, 2011; Censor et al., 2012; Winstein et al., 2014; Krakauer, 2015; Torriani-Pasin et al., 2016; Buch et al., 2017). Therefore, given its practical relevance, it seems important that motor learning studies provide information not only on the statistical significance and size, but also on the likely cause or origin of any reported changes in performance, that is, on the contribution of both real modifications in performance and random measurement error to the reported changes.

While the traditional *p*-value-based approaches for examining differences in motor performance (e.g., *t*-tests, ANOVAs, etc.) provide information on the statistical significance of a given change in performance scores, they do not inform as to the likely cause of that change. One way to address this issue and differentiate between real change and random measurement error is through the utilization of the statistics of standard error of measurement (SEM) and minimal detectable change (MDC), both of which are considered best practice in the clinical domain and therefore have been widely employed in the clinical literature (Fritz et al., 2009; Beninato and Portney, 2011; Scalzitti, 2014; Portney and Watkins, 2015). SEM is estimated from the standard deviation of a sample of scores at baseline and a test–retest reliability index of the measurement instrument or test used (e.g., SEM = s_baseline_ × ($\sqrt{1}$ - intra-class correlation coefficient (ICC); see Supplementary Material) (Beninato and Portney, 2011; Portney and Watkins, 2015). The SEM value might be considered an estimation of the expected random variation in scores when no real change has taken place (Beninato and Portney, 2011; Portney and Watkins, 2015). MDC, in turn, is estimated from SEM and a degree of confidence, usually 95% (e.g., MDC_95_ = SEM × 1.96 × $\sqrt{2}$; see Supplementary Material) (Beninato and Portney, 2011; Portney and Watkins, 2015). The MDC value might be regarded as the minimum amount of change that needs to be observed, at either the group or individual level, for it to be considered a real change (Fritz et al., 2009; Beninato and Portney, 2011; Portney and Watkins, 2015), or a change to which the contribution of real modifications in performance is likely to be greater than the contribution of random measurement error.

We designed a computer-based motor task to illustrate the applicability of SEM and MDC to motor learning research. Two studies were conducted with healthy participants. Study 1 assessed the test–retest reliability of the task and served as the basis for Study 2, which in turn consisted in a typical motor learning study, where participants practiced the task for five consecutive days. In Study 2, the data were analyzed with a traditional *p*-value-based analysis of difference (ANOVA) and also with the statistics of SEM and MDC, in order to unravel the likely origin of any changes in performance that would emerge from training.

## Materials and Methods

### Participants

Thirty-three adult individuals were recruited for Studies 1 and 2 and those who took part in one study did not participate in the other. Individuals were students or members of staff from the University of Surrey and were all right-handed. Both studies were approved by the University of Surrey’s Ethics Committee and all participants gave written consent prior to participation. Two participants withdrew from the studies (one from Study 1 and one from Study 2), leaving *N* = 16 for Study 1 and *N* = 15 for Study 2. Participation was reimbursed with £5 for Study 1 and £25 for Study 2. The latter also included a performance-related cash bonus to encourage motivation. Demographic data for both studies are presented in **Table 1**.

**Table 1:** Demographic data from the participants who completed Studies 1 and 2.

	Age (years)	Gender
Study 1 (*N* = 16)	29.38 (±8.89)	10 Females
Study 2 (*N* = 15)	20.93 (±3.22)	12 Females


*Data presented as *M* (±SD).*

### Motor Task

**FIGURE 1:**
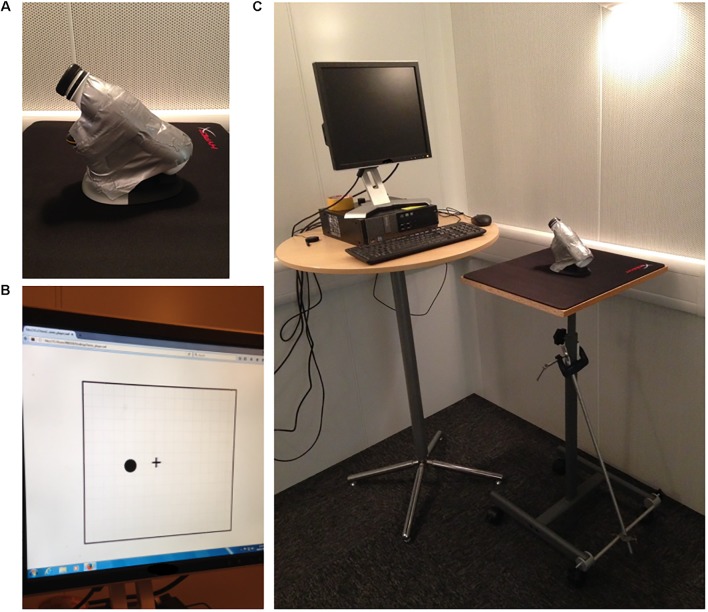
The adapted vertical mouse that had to be controlled by participants while performing the computer-based motor task during Studies 1 and 2 **(A)**, what the task consisted in **(B)**, and the experimental setup for Studies 1 and 2 **(C)**.

A computer-based motor task which involves the dexterous manipulation of an adapted vertical mouse was used in Studies 1 and 2. The adapted mouse comprised a commercially available wireless vertical mouse (Penguin Ambidextrous Vertical Mouse, Posturite Ltd., United Kingdom) with a plastic bottle attached to its vertical handle in order to increase task difficulty and counter possible familiarity with vertical mouse use (**Figure 1A**). The task was based on a freely available online game^1^ which allows customization of many of its gaming settings. Two bespoke versions of the game were programmed for Studies 1 and 2.

**FIGURE 2:**
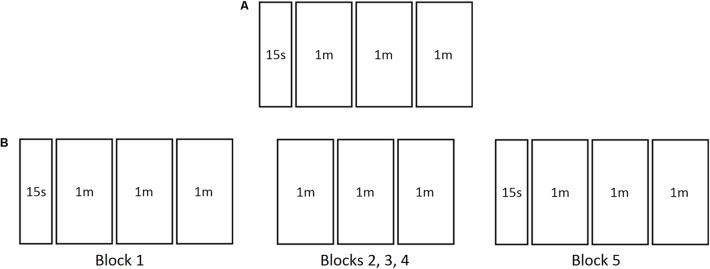
The two versions of the game used in the two present studies. The version used in Study 1 comprised only one block **(A)**, while the version used in Study 2 comprised five blocks **(B)**. 15s, 15 s familiarization trial; 1m, 1 min practice trial.

In Study 1, the game comprised one block of four trials, consisting in a 15-s familiarization trial followed by three 1-min practice trials (**Figure 2A**). A 10-s countdown preceded the familiarization trial and a 30-s countdown was given prior to commencement of every practice trial. For Study 2, the game consisted of five blocks. The first and last blocks had four trials each, as in Study 1 (one familiarization trial plus three practice trials), while the remaining blocks were comprised of three practice trials each (**Figure 2B**). A 1-min rest interval spaced blocks 1, 2, 3, and 4 from each other. Blocks 4 and 5 were spaced by a 5-min rest interval. All other parameters were similar to Study 1.

In both versions of the game, the task consisted in hitting circular targets on an 18.5 cm × 20.5 cm computer screen frame by moving a cross-hair controlled by the adapted vertical mouse (**Figure 1B**). Touching a target with the cross-hair already counted as a hit; no clicking was needed. After each target was hit, another one would immediately pop up on the screen following a pseudorandomised spatial distribution pattern. No penalty was incurred if participants missed the targets and/or moved the cross-hair outside the screen frame. However, these indirectly reduced performance because of time costs. Participants were instructed to hit as many targets as possible during the practice trials.

The adapted mouse was placed on a 50 cm × 42 cm smooth mouse pad (HyperX FURY Pro Gaming mouse pad, Kingston Technology Corporation, United States), which was fixated to a height-adjustable table (**Figure 1C**). The task was performed in a standing position in order to increase task difficulty by adding postural demands. Table height was individually adjusted so that the participants’ right upper limb would not touch the mouse pad, again to place greater demand on motor control. Individuals were instructed to keep their left upper limb hanging on the side. The cross-hair on the screen was to be controlled by sliding the mouse across the pad without lifting it. Every trial started from a central position, i.e., with the cross-hair and the mouse at the center of the computer screen frame and the pad, respectively. The apparatus was slightly shifted to the right so that participants remained aligned to the center of the computer screen (**Figure 1C**).

### Procedures

#### Study 1 (Test–Retest)

Performance was assessed on two separate days with a minimal interval of 3 days (7 days maximum; *M* = 4.5, SD = 1.37). Repeated-measures sessions (Session 1 on day 1 and Session 2 on day 2) were completed in the laboratory, with identical instructions. Sessions lasted 4 min and were controlled for time of day. No performance feedback was given after Session 1 or immediately before Session 2 to ensure the integrity of the test–retest reliability measure.

#### Study 2 (Motor Learning)

Performance was assessed on five consecutive weekdays (Sessions 1–5), with an interval of 24 h between two consecutive sessions, and after a 1-week long-term retention interval (Session 6). Before commencement of Sessions 2–5, participants were informed about their performance on the previous session; no performance feedback was given before commencement of Session 6. The game version from Study 1 (one block with four trials) was used for Session 6. Repeated-measures sessions (Sessions 1–5) were completed in the laboratory throughout the week, from Monday to Friday, with identical instructions and a 30-min duration each. One week from Friday (Session 5), another experimental session (Session 6; 4-min duration) was performed in order to assess the long-term retention of the participants’ skill on the task. All sessions were controlled for time of day.

### Data Analysis

For both studies, the number of targets hit and the average response time (RT), defined as the time in milliseconds elapsed between a target appearing on the screen and being hit, served as outcome parameters for statistical analysis.

#### Study 1 (Test–Retest)

The number of hits and the average RT from the three practice trials were averaged for each participant and session. The data were assessed for outliers and the assumption of normality. As the latter was met, paired-samples *t*-tests were then conducted to examine performance differences between the two experimental sessions. The ICC was estimated as an index of test–retest reliability, using the model of random effects and the form of single-measures, i.e., ICC (2,1) (see Supplementary Material). Alpha level was set to 5% and the Statistical Package for the Social Sciences (SPSS, version 22) was used for statistical analysis.

#### Study 2 (Motor Learning)

The number of hits and the average RT from the three practice trials from the first block were averaged for each participant and session. No outliers were identified and the assumption of normality was met. One-way repeated-measures ANOVAs, with Session as the within-subjects factor, were then conducted for both number of hits and RT data to assess performance changes across the six experimental sessions. The method of Simple Contrast was used and Session 1 was defined as the Reference Category. In order to adjust for multiple comparisons, the Bonferroni correction method was applied. When the assumption of sphericity was not met, as assessed through Mauchly’s test, the Greenhouse–Geisser correction was performed (Field, 2013). Alpha level was set to 5% and the data were handled with the SPSS (version 23).

The statistics of SEM and MDC_95_ were estimated for both hits and RT data (as described in the Supplementary Material). For estimating SEM, the standard deviation from the first block of Session 1 and the test–retest reliability index obtained in Study 1 were used (Beninato and Portney, 2011; Portney and Watkins, 2015). At the group level, for a change in performance to be considered a real change, that is, a change that is likely to be due mostly to real modifications in performance, the 95% confidence interval (CI) of the respective mean of the differences had to be outside the range of random measurement error, i.e., outside the interval spanning between the ±MDC_95_ values (Fritz et al., 2009; Beninato and Portney, 2011; Portney and Watkins, 2015). MDC_95_ proportions were also calculated. These represented the percentages of participants showing motor learning during the training period, i.e., showing an improvement in performance that was equal to or greater than the absolute values of the MDC_95_ (Portney and Watkins, 2015). By definition, changes equal to or greater than the MDC_95_ are outside the range of random measurement error and hence are likely to be caused mostly by real modifications in performance, e.g., by learning (Beninato and Portney, 2011; Portney and Watkins, 2015).

## Results

### Study 1 (Test–Retest)

The number of hits was higher in Session 2 (*M* = 78.25, SD = 7.61) than in Session 1 (*M* = 76.75, SD = 7.59). This difference, 1.50 ± 2.94, 95% CI (-0.07, 3.07), however, was not statistically significant, *t*(15) = 2.04, *p* = 0.06, and represented a small-sized effect, *d* = 0.20. A similar pattern was observed for RT, which was smaller in Session 2 (*M* = 728.24 ms, SD = 58.98) than in Session 1 (*M* = 738.86 ms, SD = 60.81). Likewise, this difference, –10.63 ± 24.11 ms, 95% CI (-23.47, 2.22), was not statistically significant, *t*(15) = 1.76, *p* = 0.098, and represented a small-sized effect, *d* = 0.18.

Estimated ICCs for number of hits and RT were 0.91, 95% CI (0.75, 0.97), *F*(15) = 25.65, *p* < 0.001, and 0.91, 95% CI (0.75, 0.97), *F*(15) = 23.69, *p* < 0.001, respectively, indicating good test–retest reliability for the task. The results are summarized in **Table 2**.

**Table 2:** Number of hits and RT data from both experimental sessions from Study 1.

Session 1	Hits	76.75 (±7.59)
	RT	738.86 ms (±60.81)
Session 2	Hits	78.25 (±7.61)
	RT	728.24 ms (±58.98)
Difference	Hits	1.50 (±2.94), 95% CI (-0.07, 3.07), *d* = 0.20
	RT	-10.63 ms (±24.11), 95% CI (-23.47, 2.22), *d* = 0.18
ICC (2,1)	Hits	0.91, 95% CI (0.75, 0.97)
	RT	0.91, 95% CI (0.75, 0.97)


*The differences between sessions and the estimated ICCs are also displayed. Data presented as *M* (±SD).*

### Study 2 (Motor Learning)

**FIGURE 3:**
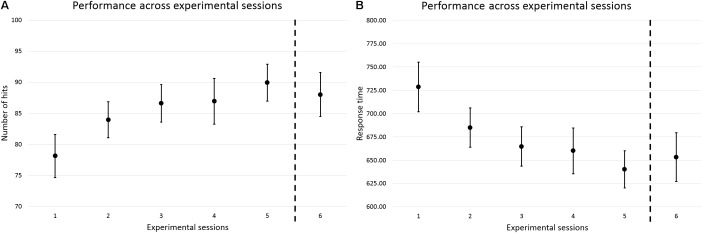
Performance across experimental sessions, in terms of number of hits **(A)** and RT **(B)**. The round dots represent the averages of the three practice trials from the first block of each experimental session, which were used to measure motor learning. The vertical dashed lines represent the 1-week long-term retention interval spanning between Sessions 5 and 6. Group data are displayed. Error bars = 95% CI.

For both parameters, participants performed increasingly better in Sessions 2–5, when compared to Session 1, and the improvements in performance achieved at the end of the training period were largely sustained over the 1-week long-term retention interval (Session 5 to 6) (**Figure 3** and **Table 3**). These observations were corroborated by a main effect of Session on number of hits and RT. *Post hoc* comparisons revealed statistically significant improvements in performance from Session 1 to 2, 1 to 3, 1 to 4, and 1 to 5, and retention from Session 5 to 6. The results are summarized in **Table 4**.

**Table 3:** Performance scores for both hits and RT data from Study 2.

Hits	Session 1	*M* = 78.11, *SD* = 6.26
	Session 2	*M* = 83.98, *SD* = 5.23
	Session 3	*M* = 86.60, *SD* = 5.42
	Session 4	*M* = 86.93, *SD* = 6.61
	Session 5	*M* = 89.93, *SD* = 5.38
	Session 6	*M* = 88.02, *SD* = 6.41

RT	Session 1	*M* = 728.69 ms, *SD* = 48.08
	Session 2	*M* = 685.02 ms, *SD* = 38.18
	Session 3	*M* = 664.76 ms, *SD* = 38.00
	Session 4	*M* = 660.04 ms, *SD* = 44.46
	Session 5	*M* = 640.16 ms, *SD* = 36.14
	Session 6	*M* = 653.35 ms, *SD* = 47.13


**Table 4:** ANOVAs and *post hoc* comparisons for both hits and RT data from Study 2.

	ANOVA	*Post hoc* comparisons	
Hits	*F*(5,70) = 17.43, *p* < 0.001	Session 1 to 2	+5.87, 95% CI (1.35, 10.39), *p* = 0.006, *r* = 0.60
		Session 1 to 3	+8.49, 95% CI (2.87, 14.11), *p* = 0.002, *r* = 0.67
		Session 1 to 4	+8.82, 95% CI (1.78, 15.87), *p* = 0.009, *r* = 0.58
		Session 1 to 5	+11.82, 95% CI (5.28, 18.37), *p* < 0.001, *r* = 0.74
		Session 5 to 6	-1.91, 95% CI (1.39, -5.22), *p* = 0.906

RT	*F*(2.64,36.95) = 18.07, *p* < 0.001	Session 1 to 2	-43.67 ms, 95% CI (-7.10, -80.24), *p* = 0.013, *r* = 0.56
		Session 1 to 3	-63.93 ms, 95% CI (-20.09, -107.78), *p* = 0.002, *r* = 0.65
		Session 1 to 4	-68.65 ms, 95% CI (-15.52, -121.78), *p* = 0.007, *r* = 0.60
		Session 1 to 5	-88.53 ms, 95% CI (-39.22, -137.84), *p* < 0.001, *r* = 0.74
		Session 5 to 6	+13.19 ms, 95% CI (-12.11, 38.49), *p* = 1.00


**FIGURE 4:**
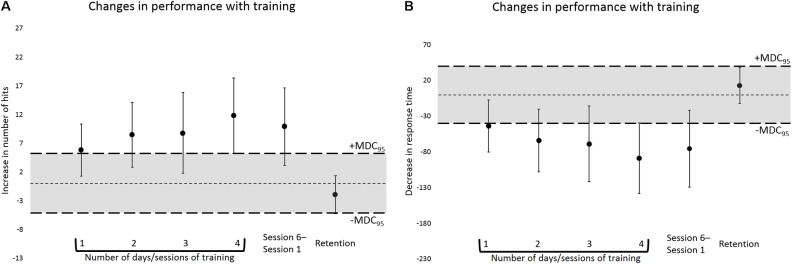
Changes in performance, in terms of number of hits **(A)** and RT **(B)**, as a function of the number of days/sessions of training on the task. From left to right: Means of the differences in performance between Sessions 1 and 2, 1 and 3, 1 and 4, 1 and 5, 1 and 6, and 5 and 6. The thin dashed horizontal lines indicate a mean difference of 0. The thick dashed horizontal lines indicate the ±MDC_95_ values (±5.21 for number of hits and ±39.97 ms for RT). The shaded areas between the ±MDC_95_ values represent the interval corresponding to the range of random measurement error. Changes in performance that are within this interval, irrespectively of statistical significance, are likely to result mostly from random measurement error, as opposed to from real modifications in performance, e.g., from learning. Group data are displayed. Error bars = 95% CI.

**FIGURE 5:**
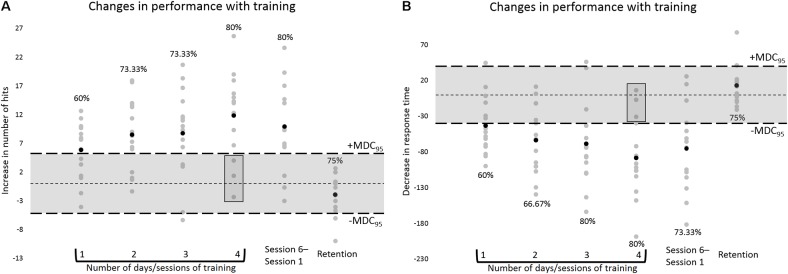
The same type of data as in **Figure 4** are displayed, but here data are displayed at the individual level. The percentages represent the MDC_95_ proportions. These values correspond to the percentages of participants showing motor learning during the training period, i.e., showing an improvement in performance that was equal to or greater than the absolute values of the MDC_95_ and that therefore was outside the range of random measurement error, and hence was likely to have been caused mostly by learning. The rectangular boxes highlight the participants displaying a change in performance at the end of the training period, i.e., after 4 days/sessions of training, that was smaller than the absolute values of the MDC_95_. These individuals could be considered “non-learners.” The percentages at Retention represent the percentages of participants who, having displayed motor learning at the end of the training period, showed a change in performance at the 1-week long-term retention test (Session 5 to 6) that was smaller than the absolute values of the MDC_95_ and that therefore was likely to have been caused mostly by random measurement error.

The estimates of SEM and MDC_95_ were, respectively, 1.88 and 5.21 for number of hits, and 14.42 and 39.97 ms for RT. At the group level, for both number of hits and RT, from 1 to 4 days/sessions of training on the task, the 95% CI of the mean of the differences in performance moved away from the interval corresponding to the range of random measurement error, suggesting a trend toward a real improvement in performance with training, i.e., learning (please refer to **Figure 4**). Analysis of individual data confirmed this trend by showing an increase in the MDC_95_ proportion (60–80%) from 1 to 4 days/sessions of training, for both number of hits and RT, hence indicating a motor learning effect for individual participants (please refer to **Figure 5**).

At the 1-week long-term retention test, at the group level, for both number of hits and RT, the 95% CI of the mean of the differences in performance between Sessions 5 and 6 was within the interval corresponding to the range of random measurement error (please refer to **Figure 4**). At that same time point, 75% of the participants (9 out of 12) who after 4 days/sessions of training displayed learning, i.e., an improvement in performance that was equal to or greater than the absolute values of the MDC_95_, showed a change in performance which was within the range of random measurement error (please refer to **Figure 5**). Altogether, these findings indicate good long-term retention of motor learning from Session 5 to 6.

**Table 5** displays the estimates of SEM and MDC_95_ for both the number of hits and RT data. The respective MDC_95_ proportions at the end of the training period are also displayed.

**Table 5:** Statistics of SEM and MDC_95_ for both number of hits and RT data from Study 2.

	SEM	MDC_95_	MDC_95_ proportion
Hits	1.88	5.21	80%
RT	14.42 ms	39.97 ms	80%


*The respective MDC_*95*_ proportions at the end of the training period, i.e., after 4 days/sessions of training on the task, are also displayed.*

## Discussion

Motor learning studies face the challenge of differentiating between real changes in performance and random measurement error. One way of meeting that challenge is through the utilization of the statistics of SEM and MDC. The MDC value represents the minimum amount of change that needs to be observed for it to be considered a real change, or a change that exceeds random measurement error and therefore is likely to be produced mostly by real modifications in performance. We designed a computer-based motor task to illustrate the applicability of SEM and MDC to motor learning research. Two studies were conducted with healthy participants. Study 1 assessed the test–retest reliability of the task and Study 2 consisted in a typical motor learning study, where participants practiced the task for five consecutive days. In Study 2, the data were analyzed with a traditional *p*-value-based analysis of difference (ANOVA) and also with SEM and MDC, in order to determine the likely cause of any changes in performance emerging from training.

Overall, the results showed that our task is reliable and that the *p*-value-based analysis alone identified statistically significant improvements in performance over time even when the observed changes could in fact have been smaller than the MDC and thereby caused mostly by random measurement error, as opposed to by motor learning.

### Study 1 (Test–Retest)

According to general guidelines (e.g., Portney and Watkins, 2015), our results suggest good test–retest reliability for the computer-based motor task we designed (**Table 2**). Although performance improved slightly from test (Session 1) to retest (Session 2), these differences were not statistically significant and represented small effects and, more importantly, did not affect test–retest reliability (**Table 2**).

Ensuring the test–retest reliability of a task/test not only gives researchers more confidence for attributing potential changes in performance scores to an experimental manipulation but, critically, it also allows for the estimation of the statistics of SEM and MDC, which in turn can contribute to unravel the likely origin of those changes (Beninato and Portney, 2011; Portney and Watkins, 2015).

### Study 2 (Motor Learning)

Motor learning was assessed through four consecutive short-term retention tests administered 24 h after completion of the preceding training session. Such tests corresponded to the first block of the second, third, fourth, and fifth experimental sessions (**Figure 3**). It has been suggested that 24 h since the last practice session is an adequate minimal interval for the application of retention tests and thereby the assessment of motor learning (see Kantak and Winstein, 2012 for further discussion). The long-term retention of motor learning after 5 days/sessions of training on the task was also investigated through another retention test which was administered 1 week after the last training day/session. This test corresponded to the sixth experimental session (**Figure 3**).

At the group level, according to the *p*-value-based analysis of difference, for both hits and RT data, motor learning took place as early as after 1 day/session of training (Session 1 to 2), with improvements in performance increasing with the number of days/sessions of training (**Figure 4** and **Table 4**). The analyses also showed good long-term retention of motor learning 1 week after the end of the training period (**Figure 4** and **Table 4**). However, the analyses based on SEM and MDC revealed a somewhat different scenario. For instance, after 1 day/session of training on the task (Session 1 to 2), both in terms of number of hits and RT, the 95% CI of the mean of the differences in performance overlapped largely with the interval corresponding to the range of random measurement error, that is, the interval spanning between the respective ±MDC_95_ values (**Figure 4** and **Table 4**). This means that, at that stage, the respective improvements in performance could in fact have been caused mostly by random measurement error, as opposed to by learning. The scenario remained rather similar as training progressed to 2 and 3 days/sessions of training on the task. It was only at the end of the training period, i.e., after 4 days/sessions of practice, that the 95% CI of the mean of the differences practically did not overlap anymore with the interval corresponding to the range of random measurement error—there was no overlap for the hits data and the overlap was minimum for RT (**Figure 4** and **Table 4**). This indicates therefore that it was only at that stage that the respective improvements in performance (Session 1 to 5) were more likely to reflect real improvements, i.e., to have been caused mostly by motor learning, as opposed to by random measurement error. This observation is critical as it could be used to inform future studies as to the minimum amount of training on the task that would be needed in order to produce robust motor learning. For instance, instead of only 1 or 2, or even 3 days/sessions of training, as suggested by the *p*-value-based analyses alone, adopting a longer training regime, e.g., at least 4 days/sessions of practice, would be a more appropriate conduct. By doing so, one could then be more confident that motor learning would be contributing more than random measurement error to any observed improvements in performance.

This seeming discrepancy between *p*-value- and MDC-based analyses might be due to the fact that the former does not take into account the random error that is associated with the measurement instruments producing the means which in turn are compared by the traditional inferential statistical tests, while the latter is performed directly from an estimation of the size of that error (see the respective formulas in the Supplementary Material). Assuming that measures of motor performance and their change over time are free from random error, irrespectively of statistical significance, is at odds with good research practice and should be avoided. This is a fundamental issue for motor learning studies, which typically test for improvements in performance scores from pre- to post-training assessments. In order to further support our abovementioned findings and arguments, by using the data obtained in Study 1, we simulated 10 hypothetical motor learning studies on a freely available statistical software (ESCI^2^ – Chapters 5 and 6, 2011; see also Cumming, 2012, 2014 for further information on ESCI). Details of how these simulations were performed and their results are available in the Supplementary Material. In short, these simulated studies compared performance scores between a pre- and a post-training assessment, and they all produced two-tailed *p*-values < 0.05 for a paired-samples *t*-test with α = 0.05, and effect sizes varying from medium to large (minimum of 0.49 and maximum of 0.95). Although all simulated studies yielded statistically significant results and medium-to-large effect sizes, hence suggesting improvements in performance, i.e., learning, from the pre- to the post-training assessments, in 9 out of the 10 studies the 95% CI of the mean of the differences in performance scores overlapped with the interval corresponding to the range of random measurement error. In four studies, the overlap was total. In only 1 out of the 10 studies the 95% CI of the mean of the differences was completely outside (above) the interval of random measurement error —this particular result mirrors our abovementioned result for the improvement in performance, in terms of number of hits, that took place after 4 days/sessions of training on our task (Session 1 to 5). Overall, the results from these simulated studies lend strong support to the argument that *p*-value-based analyses of difference alone do not inform as to the likely origin of changes in performance scores, and that even improvements in performance which are found to be statistically significant can sometimes be due mostly to random measurement error, instead of to motor learning.

During the analysis of individual data, for both number of hits and RT, from 1 to 4 days/sessions of training on the task, an increase in the MDC_95_ proportion was observed (60–80%) (**Figure 5**), indicating a motor learning effect for some participants at the individual level. Nevertheless, at the end of the training period, i.e., after 4 days/sessions of practice, for both hits and RT, that proportion did not reach 100%. This was due to the fact that, at that stage, 20% of the participants (3 out of 15) displayed a change in performance which was smaller than the absolute values of the MDC_95_ (**Figure 5**). These participants could be considered “non-learners,” as their respective change in performance was likely to have been caused mostly by random measurement error, and not by learning. People differ in the way they respond to training and although we did not formally address this issue, it could be speculated for instance that such individual differences in learning might have emerged from variations in people’s motor ability, which according to Schmidt and Lee (2014) may be defined as “a fundamental characteristic of different individuals that tends to underlie particular skills; ability is largely inherited genetically and is not modifiable by practice” (p. 190).

## General Discussion and Conclusion

Motor training or practice leads to motor learning, that is, to relatively permanent or stable improvements in motor performance (Schmidt and Lee, 2014). It has been suggested that learning a motor task, including tasks requiring the control of tools such as a computer mouse, involves developing and optimizing internal models of that task (Wolpert et al., 2001, 2011; Wise and Willingham, 2009). Internal models represent sensorimotor transformations in the brain, i.e., mappings between motor commands and their sensory consequences. Therefore, when learning how to control a device such as a computer mouse, individuals develop and optimize mappings between their actions on the device and the consequences that are generated, in this case on the computer screen (see Imamizu et al., 2000; Imamizu and Kawato, 2012 for further discussion). Although this was not formally addressed in our studies, these model-based mechanisms could in theory explain the learning that most participants exhibited in Study 2. It is possible though, that model-free learning mechanisms, including use-dependent plasticity, operant reinforcement and/or success-based exploration might also have played a role (Krakauer and Mazzoni, 2011).

While extensively acknowledged in the clinical literature (Fritz et al., 2009; Beninato and Portney, 2011; Scalzitti, 2014; Portney and Watkins, 2015), the issue of differentiating between real changes and random measurement error, and hence the use and reporting of statistics such as SEM and MDC, has not received much attention in motor learning research. Here we have shown the applicability of these statistical concepts to the context of motor learning, and how their utilization might contribute to determine the likely cause of changes in performance that normally occur in response to training. For instance, the use of the MDC statistics, which in turn is estimated from SEM, allows for differentiating between real modifications in performance and random measurement error. The MDC value might be regarded as the minimum amount of change that needs to be observed, at either the group or individual level, for it to be considered a real change (Fritz et al., 2009; Beninato and Portney, 2011; Portney and Watkins, 2015), or a change to which the contribution of real modifications in performance is likely to be greater than the contribution of random measurement error. Measurement instruments, including the tests or tasks that are commonly used in motor learning research, are not free from random error. Finding statistically significant differences between pre- and post-training assessments that suggest improvements in performance, i.e., motor learning, does not preclude the possibility that such differences are being produced mostly by random measurement error, as opposed to by learning. Moreover, *p*-value-based analyses of difference (e.g., *t*-tests, ANOVAs, etc.) tend to focus on group changes while ignoring what is happening at the individual level. Inter-individual variability has been a major problem in both the motor learning (Schmidt and Lee, 2014) and the plasticity and brain stimulation arenas (Ridding and Ziemann, 2010; Di Lazzaro and Rothwell, 2014; Buch et al., 2017). Being able to identify and dissociate “learners” from “non-learners,” or “responders” from “non-responders” in the case of brain stimulation-based motor learning investigations, for instance (e.g., Buch et al., 2017), might contribute to better elucidate both the intervention’s mechanisms and the factors mediating inter-individual differences. As we have shown here, the use and reporting of the statistics of SEM and MDC might be an interesting approach to addressing these issues.

We suggest that motor learning studies could complement their *p*-value-based analyses of difference with statistics such as SEM and MDC in order to inform as to the contribution of both real modifications in performance and random measurement error to any reported changes in motor performance.

## Author Contributions

LF designed the work, collected, analyzed, and interpreted the data, and wrote the first draft and subsequent versions of the manuscript. AS provided intellectual contribution to the design of the work, critically revised the first draft and the subsequent versions of the manuscript, and approved the final version of the text to be published.

## Conflict of Interest Statement

The authors declare that the research was conducted in the absence of any commercial or financial relationships that could be construed as a potential conflict of interest.

## Footnotes

1 www.aimbooster.com

2 http://thenewstatistics.com/itns/esci/esci-for-utns/
