# Supplementary material for: The Applicability of Standard Error of Measurement and Minimal Detectable Change to Motor Learning Research—A Behavioral Study
Source: Front Hum Neurosci. 2018 Mar 22;12:95. doi: 10.3389/fnhum.2018.00095 (PMC5875129; doi:10.3389/fnhum.2018.00095)
Supplement: Supplementary file 1 [file Data_Sheet_1.docx]

Supplementary Material

**The applicability of Standard Error of Measurement (SEM) and Minimal Detectable Change (MDC) to motor learning research – a behavioural study**

Leonardo Furlan^1^, Annette Sterr^1,2^*

1- Brain and Behaviour Research Group, School of Psychology, Faculty of Health and Medical Sciences, University of Surrey, Guildford, Surrey, UK.

2- Neurology Clinical Division, Clinics Hospital, São Paulo University, São Paulo, SP, Brazil.

*** Correspondence:** Annette Sterr ([a.sterr@surrey.ac.uk](mailto:a.sterr@surrey.ac.uk))

# Supplementary Data

1) Intra-class Correlation Coefficient (ICC)

The ICC (2,1) was estimated by SPSS (version 22). Its formula is as follows:

**ICC (2,1) = (BMS – EMS) / ((BMS + (k – 1) x EMS) + (k x (RMS – EMS) / n))**

Where BMS corresponds to the between-subjects variance, EMS to the error variance, RMS to the between sessions variance, k to the number of sessions or testing conditions, and n to the sample size.

2) Standard Error of Measurement (SEM) and Minimal Detectable Change (MDC_95_)

The SEM was calculated as follows: **SEM = s_baseline_ x √(1 – ICC)**

Where s_baseline_ corresponds to the standard deviation of a baseline session/test.

The MDC_95_ was calculated as follows: **MDC_95_ = SEM x 1.96 x √2**

Where 1.96 corresponds to the level of confidence adopted (in this case, 95%) and √2 represents a correction factor for repeated measurements.

**-MDC_95_ < Δ < +MDC_95_**

Δ = Change due mostly to random measurement error

**-MDC_95_ > Δ > +MDC_95_**

Δ = Change due mostly to real modifications in performance, e.g., learning (real change)

Ten hypothetical motor learning studies were simulated on a freely available statistical software (ESCI - Chapters 5 and 6, 2011; <http://thenewstatistics.com/itns/esci/esci-for-utns/>; see also (Cumming, 2012, 2014) for further information on ESCI), in which individuals were assessed for their performance before and after a hypothetical period of training. In other words, 10 “pre- and post-training assessment” studies were simulated. Data from our Study 1 were used to obtain some statistics for the simulations (**Supplementary** **Table 1**). These included the standard deviations of the means from both experimental sessions (SD_Session1_ and SD_Session2_) and the standard deviation of the mean of the differences between sessions (s_diff_). In order to run the simulations, these values were used as estimates for the corresponding parameters in the population, i.e., to estimate σ (the population’s standard deviations of the means, assumed here to be the same for the pre- and post-training assessments) and σ_diff_ (the population’s standard deviation of the mean of the differences). With regards to the population’s means for the pre- and post-training assessments (µ_Pre-training_ and µ_Post-training_, respectively), these values were chosen so as to produce a mean of the differences in the population corresponding to a medium-to-large effect size. In order to meet that requirement, a value of 5 for the population’s mean of the differences was chosen. The sample size for all the 10 simulated studies was the same as that from our Study 1 (N = 16). After determining all of the aforementioned parameters (**Supplementary** **Table 1**), the 10 simulations of motor learning studies were then performed on the statistical software. The **Supplementary** **Figure** displays the output from the software for one of the simulations. After simulating the 10 studies and recording their results, the statistics of SEM and MDC_95_ were estimated for each study. For each simulated study, for estimating SEM, the standard deviation from the respective pre-training assessment and the test-retest reliability index obtained in our Study 1, i.e., ICC (2,1), were used (Beninato & Portney, 2011; Portney & Watkins, 2015).

The simulated studies, along with their results and respective SEM and MDC_95_ estimates, are displayed in **Supplementary** **Table 2**. The population parameters that were estimated and used for the simulations (**Supplementary** **Table 1**) corresponded to a medium-to-large effect size in the population (Cohen’s δ = 0.63, automatically estimated by ESCI). All simulated studies produced two-tailed p-values < .05 for a paired-samples t-test with α = .05. Effect sizes, as indexed through Cohen’s d_unb_ (unbiased version of Cohen’s d, automatically estimated by ESCI), varied from medium to large (minimum of 0.49 and maximum of 0.95).

Although all simulated studies yielded statistically significant results and medium-to-large effect sizes, hence suggesting improvements in performance, i.e., learning from the pre- to the post-training assessments, in 9 out of the 10 studies the 95% CI of the mean of the differences in performance scores overlapped with the interval corresponding to the range of random measurement error, that is, the interval spanning between the respective ±MDC_95_ values. In 4 studies the overlap was total. In only 1 out of the 10 studies the 95% CI of the mean of the differences was completely outside (above) the interval of random measurement error. These results show that p-value-based analyses of difference alone do not inform as to the likely cause or origin of changes in performance scores, and that even improvements in performance which are found to be statistically significant can sometimes be due mostly to random measurement error, instead of to motor learning.

**References**

Beninato, M., & Portney, L. G. (2011). Applying concepts of responsiveness to patient management in neurologic physical therapy. *Journal of Neurologic Physical Therapy*, *35*(June), 75–81. https://doi.org/10.1097/NPT.0b013e318219308c

Cumming, G. (2012). *Understanding the new statistics: Effect sizes, confidence intervals, and meta-analysis*. New York: Routledge. https://doi.org/10.1037/a0028079

Cumming, G. (2014). The new statistics: Why and how. *Psychological Science*, *25*(1), 7–29. https://doi.org/10.1177/0956797613504966

Portney, L., & Watkins, M. (2015). *Foundations of Clinical Research : Applications to Practice* (3rd Editio). FADavis.

# Supplementary Figure and Tables


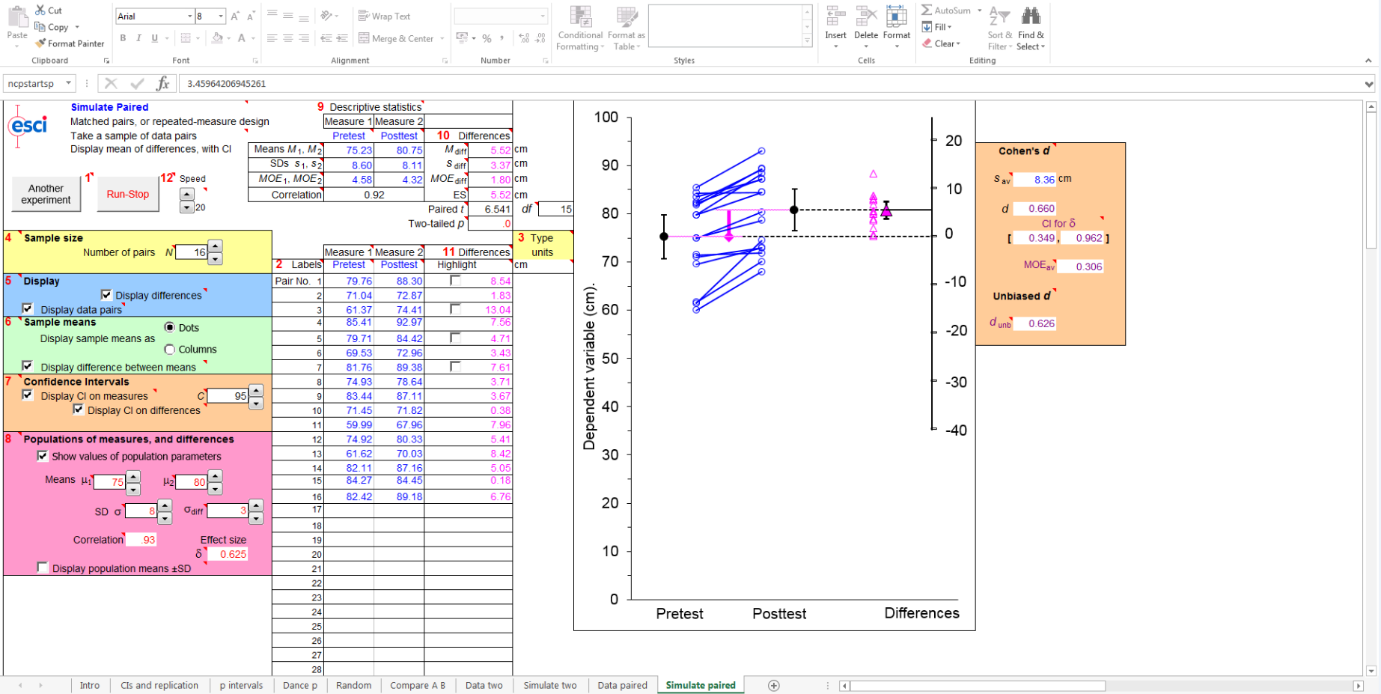


**Supplementary Figure.** Display of the output from ESCI for one of the simulated studies.

**Supplementary Table 1.** Data used for the simulations. See text for further details.

| SD_Session1_ | Obtained from Study 1 | 7.59 |
| --- | --- | --- |
| SD_Session2_ |  | 7.61 |
| s_diff_ |  | 2.94 |
| µ_Pre-training_ | Estimated population parameters | 75 |
| µ_Post-training_ |  | 80 |
| σ |  | 8 |
| σ_diff_ |  | 3 |

**Supplementary Table 2.** Characteristics and results from the 10 simulated hypothetical motor learning studies. In red are the studies where the 95% CI of the mean of the differences in performance scores between the pre- and post-training assessments overlapped with the interval corresponding to the range of random measurement error. In bold red are the studies where that overlap was total. In blue is the only one study where the 95% CI of the mean of the differences was completely outside (above) the interval of random measurement error, and therefore where the improvement in performance from the pre- to the post-training assessment was more likely to have been caused mostly by learning, as opposed to by random measurement error. M = Mean; SD = Standard Deviation.

| Simulated  Studies |  | Pre-training | Post-training | Difference | Two-tailed p | d_unb_ | SEM | MDC_95_ |
| --- | --- | --- | --- | --- | --- | --- | --- | --- |
| Study  1 | M | 75.23 | 80.75 | 5.52,  95% CI [3.72, 7.32] | < .05 | 0.63 | 2.58 | 7.15 |
|  | SD | 8.60 | 8.11 | 3.37 |  |  |  |  |
| Study  2 | M | 79.66 | 84.97 | 5.31,  95% CI [3.94, 6.68] | < .05 | 0.64 | 2.42 | 6.71 |
|  | SD | 8.07 | 7.56 | 2.57 |  |  |  |  |
| Study  3 | M | 73.70 | 80.44 | 6.74,  95% CI [5.43, 8.05] | < .05 | 0.95 | 1.94 | 5.38 |
|  | SD | 6.45 | 7.05 | 2.45 |  |  |  |  |
| Study  4 | M | 72.91 | 78.78 | 5.86,  95%CI [4.48, 7.24] | < .05 | 0.84 | 2.13 | 5.90 |
|  | SD | 7.11 | 6.14 | 2.58 |  |  |  |  |
| Study  5 | M | 74.39 | 79.16 | 4.76,  95% CI [3.07, 6.45] | < .05 | 0.56 | 2.25 | 6.24 |
|  | SD | 7.50 | 8.73 | 3.17 |  |  |  |  |
| Study  6 | M | 75.10 | 79.15 | 4.05,  95% CI [2.75, 5.35] | < .05 | 0.62 | 1.90 | 5.27 |
|  | SD | 6.32 | 6.04 | 2.44 |  |  |  |  |
| Study  7 | M | 72.13 | 75.97 | 3.83,  95% CI [1.64, 6.02] | < .05 | 0.50 | 1.97 | 5.46 |
|  | SD | 6.56 | 7.96 | 4.11 |  |  |  |  |
| Study  8 | M | 78.52 | 83.61 | 5.09,  95% CI [3.13, 7.05] | < .05 | 0.56 | 2.64 | 7.32 |
|  | SD | 8.81 | 8.30 | 3.67 |  |  |  |  |
| Study  9 | M | 71.37 | 76.05 | 4.68,  95% CI [3.11, 6.25] | < .05 | 0.54 | 2.47 | 6.85 |
|  | SD | 8.22 | 8.34 | 2.94 |  |  |  |  |
| Study  10 | M | 73.76 | 78.69 | 4.93,  95% CI [3.28, 6.58] | < .05 | 0.49 | 2.97 | 8.23 |
|  | SD | 9.91 | 9.33 | 3.10 |  |  |  |  |
